# Supplementary material for: Polymerase delta-interacting protein 38 (PDIP38) modulates the stability and activity of the mitochondrial AAA+ protease CLPXP
Source: Commun Biol. 2020 Nov 6;3:646. doi: 10.1038/s42003-020-01358-6 (PMC7647994; doi:10.1038/s42003-020-01358-6)
Supplement: Supplementary file 1 — Supplementary Information [file 42003_2020_1358_MOESM1_ESM.pdf]

Polymerase delta-interacting protein 38 (PDIP38) modulates the stability and activity of the mitochondrial AAA+ protease CLPXP

Philip R. Strack[1], Erica J. Brodie[1,§], Hanmiao Zhan[1], Verena J. Schuenemann[2,†], Liz J. Valente[1,ƒ], Tamanna Saiyed[1], Brad R. Lowth[1], Lauren M. Angley[3], Matthew A. Perugini[1,3], Kornelius Zeth[2,4], Kaye N. Truscott[1*] and David A. Dougan[1*]

[1]Department of Biochemistry and Genetics, La Trobe Institute for Molecular Science, La Trobe University, Melbourne, Victoria, 3086, Australia.

[2]Department of Protein Evolution, Max Planck Institute for Developmental Biology, Tübingen, D-72076, Germany.

[3]Department of Biochemistry and Molecular Biology, The University of Melbourne, Parkville, Victoria; 3010, Australia.

[4]Department of Science and Environment, Roskilde University, DK-4000, Denmark.

[*]These authors contributed equally to this work

[§]Present address: CSL Behring, Broadmeadows, Victoria; 3047, Australia.

[†]Present address: Institute of Evolutionary Medicine, University of Zurich, Zurich, Switzerland.

[ƒ]Present address: Department of Radiation Oncology, Stanford University School of Medicine, Stanford, CA 94305, USA.

Correspondence and requests for materials should be addressed to K.N.T. (email: k.truscott@latrobe.edu.au) or D.A.D. (email: d.dougan@latrobe.edu.au).

Supplementary Table 1 Conserved residues that line and cap the hydrophobic groove

Human PDIP38 (PDB: 6ZLX)	Human Fbxo3 (PDB: 5HDW)	*X. axonopodis* ApaG (PDB: 2F1E)
Y 265	Y 308	Y 33
R 282	R 329	R 50
W 284	W 331	W 52
I 286	I 333	I 54
E 294	E 341	E 62
V 296	V 343	V 64
V 301	V 348	V 69
V 302	V 349	V 70
Y 317	Y 363	Y 84
V 321	T 367	V 88
M 330	M 376	M 97

Supplementary Table 2 Oligonucleotide primers used in this study

Primer	DNA sequence (5'-> 3')	Gene	Features
5pdip_sac2	GACTCTCCGCGGTGGATCCTCCCGAAACCGACCAGAGGGC	PDIP38	Sac II restriction site for cloning into pHUE
3pdip_hind	GCTACGAAGCTTCTACCAGTGAAGGCCTGAGGGTGG	PDIP38	Hind III restriction site for cloning into pHUE or pDD173 (to generate His$_6$-GFP-PDIP38)
5pdip_not	GCAGTAGCGGCCGCATCGTCCCGAAACCGACCAGAG	PDIP38	Not I restriction site for cloning into pET10N or pDD173 (to generate His$_6$-GFP-PDIP38)
5not_Cdip	GCAGTAGCGGCCGCACGGGAAACAACTGAGAACATACG	PDIP38$_C$	Not I restriction site for cloning into pET10N
3pdip_xho	GATAGCCTCGAGCTACCAGTGAAGGCCTGAGGGTGG	PDIP38<br>PDIP38$_C$	Xho I restriction site for cloning into pET10N or pGEX4T
5pdip_bam	GTCGATGGATCCTCCCGAAACCGACCAGAGGGC	PDIP38	Bam HI restriction site for cloning into pGEX4T
Ndip_3not	ACGTAGCGCGGCCGCATGAACATCGGAGAGCTCCAG	PDIP38$_N$	Not I restriction site for cloning into pET10C
5pdip_nde	CGTATCCATATGTCCTCCCGAAACCGACCAGAGGGC	PDIP38<br>PDIP38$_N$	Nde I restriction site for cloning into pET10C
3pdip_not	GATAGCTGCGGCCGCCCAGTGAAGGCCTGAGGGTGG	PDIP38<br>PDIP38$_C$	Not I restriction site for cloning into pET10C
3dip_STOP	GATAGCTGCGGCCGCCTACCAGTGAAGGCCTGAGGGTGG	PDIP38	Not I restriction site for cloning into pET10C (no His tag)
Ndip_3xho	ACGTAGCTCGAGCTAATGAACATCGGAGAGCTCCAG	PDIP38$_N$	Xho I restriction site for cloning into pGEX4T
PDIP_bam1	CTCAGACAGAATAAGGATCCTTCTTGGCTAACCATG	PDIP38$_N$<br>PDIP38$_C$	Introduce stop codon and Bam HI restriction site to create PDIP38$_N$ or PDIP38$_C$ in pGEX4T
PDIP_bam2	GCCAAGAAGGATCCTTATTCTGTCTGAGATCTCTGAG	PDIP38$_N$<br>PDIP38$_C$	Introduce stop codon and Bam HI restriction site to create PDIP38$_N$ or PDIP38$_C$ in pGEX4T
5prePDIP_hind3	TCGTAGAAGCTTATGGCAGCCTGTACAGCCCGGC	PDIP38	Hind III restriction site for cloning into pEGFP-N1
38G2	CGGTGGATCCCAGTGAAGGCCTGAGGGTG	PDIP38	Bam HI restriction site for cloning into pEGFP-N1
hX4A_1	CTTTGTAGCTGCAGCCGCTTTTGTCAAGTGTGAAAAG	CLPX<br>CLPX$_N$	Introduce Pst I restriction site (when converting wild type CLPX to CLPX$_{AAAA}$
hX4A_2	CTTGACAAAAGCGGCTGCAGCTACAAAGGTCTCTAC	CLPX<br>CLPX$_N$	Introduce Pst I restriction site (when converting wild type CLPX to CLPX$_{AAAA}$
Sac2_TRALP	CGAATTCCGCGGTGGAACCCGGGCTCTCCCGCTCATTC	CLPP	Sac II restriction site for cloning into pHUE
LhP_hind3	GCTACGAAGCTTAGGTGCTAGCTGGGACAGGTTC	CLPP	Hind III restriction site for cloning into pHUE

[1] restriction sites for cloning and/or screening are underlined

Supplementary Table 3 Plasmids used in this study

**Plasmid name**	**Plasmid description**	**Plasmid features, source**
pDT1329	pOTB7/*PDIP38*	I.M.A.G.E. clone 3349399
pDT1432	pHUE/PDIP38	Amplified *PDIP38* using 5pdip_sac2 and 3pdip_hind, digested with *Bam* HI and *Hind* III and cloned into pHUE
pDT1355	pET10N/*PDIP38*	Amplified *PDIP38* using 5pdip_not and 3pdip_xho, digested with *Not* I and *Xho* I and cloned into pET10N
pDT1562	pET10C/*PDIP38*	Amplified *PDIP38* using 5pdip_nde and 3pdip_not, digested with *Nde* I and *Not* I and cloned into pET10C
pDT1586	pET10C/*PDIP38*	Amplified untagged *PDIP38* using 5pdip_nde and 3dip_STOP, digested with *Nde* I and *Not* I and cloned into pET10C
pDT1356	pGEX-4T/*PDIP38*	Amplified *PDIP38* using 5pdip_bam and 3pdip_xho, digested with *Bam* HI and *Xho* I and cloned into pGEX-4T
pDT1367	pGEX-4T/$PDIP38_N$	Quick change mutagenesis using pDT1356 and primers PDIP_bam1 and PDIP_bam2
pDT1362	pGEX-4T/$PDIP38_C$	Quick change mutagenesis using pDT1356 and primers PDIP_bam1 and PDIP_bam2, digestion with *Bam* HI to remove the fragment coding for $PDIP38_N$ followed by ligation of digested plasmid
pDT2191	pE-FLAG/PDIP38-FLAG	Amplified *PDIP38* using 5prePDIP_hind3 and 38G2, digested with *Hind* III and *Bam* HI and cloned into pE-FLAG
pDT1766	pDD173/H_6GFP-PDIP38	Amplified *PDIP38* using 5pdip_not and 3pdip_hind, digested with *Not* I and *Hind* III and cloned into pDD173 (Dougan et al., 2003) (digestion with *Not* I and *Hind* III)
pDT1279	pET10C/*CLPX*	Lowth et al., 2012
pDT1255	pET10C/$CLPX_{ZBD}$	Lowth et al., 2012
pDT1260	pET10C/$CLPX_E$	Lowth et al., 2012
pDT1411	pET10C/$CLPX_{WB}$	Lowth et al., 2012
pDT1973	pET10C/$CLPX_{4A}$	Quick change mutagenesis using pDT1279 and primers hX4A_1 and hX4A_2
pDT1977	pET10C/ZBD_{4A}	Quick change mutagenesis using pDT1255 and primers hX4A_1 and hX4A_2
pDT1668	pUHIS/*CLPP*	Lowth et al., 2012
pDT2772	pHUE/*CLPP*	Bezawork-Geleta et al., 2014
pDD795	pC10HIS/ec $clpX_{ZBD}$	Dougan et al., 2003

Supplementary Figures

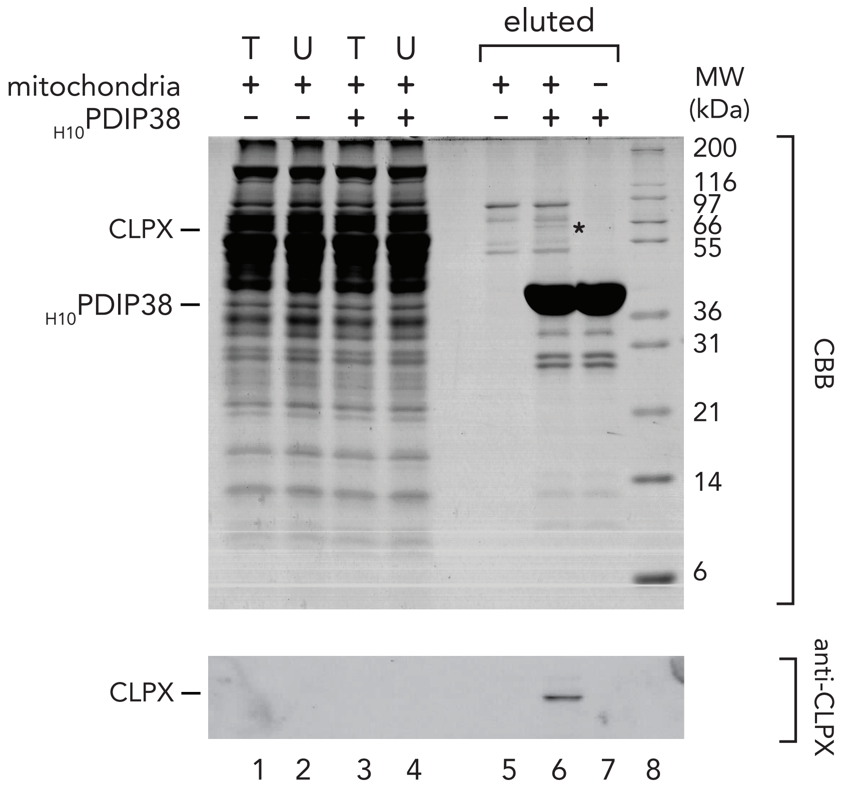

Supplementary Figure 1. In vitro interaction of recombinant H_{10}PDIP38 with mitochondrial CLPX.

Coomassie Brilliant Blue (CBB) stained polyacrylamide gel (upper panel) or anti-CLPX Western blot (lower panel) of samples from pull-down assay of mitochondrial proteins isolated from HeLa cells, using beads either lacking (lanes 1, 2 and 5) or containing immobilised purified recombinant H_{10}PDIP38 (lanes 3, 4 and 6). As an additional control immobilised H_{10}PDIP38 was incubated with buffer (lane 7), T, total (lanes 1 and 3); U, unbound (lanes 2 and 4), and eluted (lanes 5 – 7) fractions. Lane 8, See Blue Plus MW protein standards.

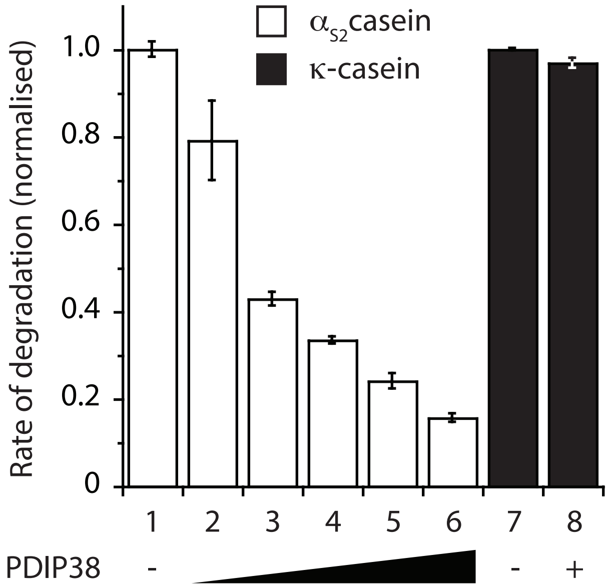

Supplementary Figure 2. The in vitro degradation of FITC-α_{S2}-casein by CLPXP is inhibited by PDIP38.

The rate of FITC-α_{S2}-casein degradation (white bars) was determined in the absence (column 1) or presence of increasing concentrations of PDIP38 [1.2 µM (column 2), 4.8 µM (column 3), 9.6 µM (column 4), 19.2 µM (column 5), 38.4 µM (column 6)]. The rate of FITC-κ-casein degradation (black bars) was determined in the absence (column 7) or presence of 38.4 µM PDIP38 (column 8).

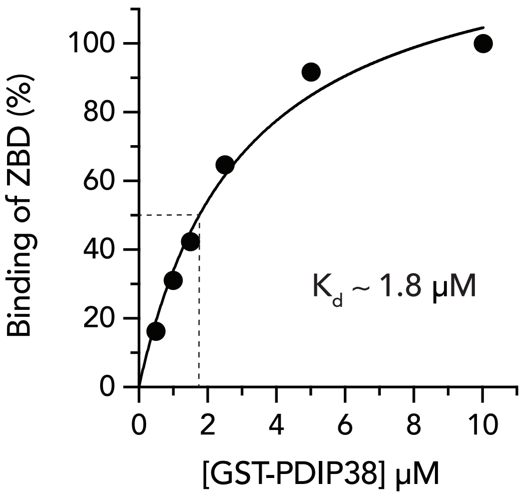

Supplementary Figure 3. Binding affinity of Human CLPX ZBD to GST-PDIP38.

The apparent dissociation constant (K_d) of human CLPX ZBD for GST-PDIP38 as determined from binding of GST-PDIP38 (500 nM – 10 µM) to immobilised ZBD.

a

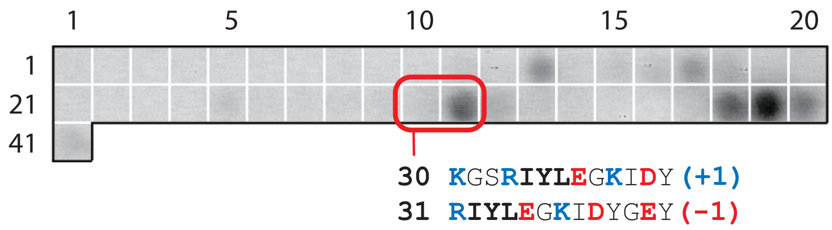

b

spot	aa	sequence	spot	aa	sequence
1	1	ESETTTSLVLERS	21	61	DVSQKTTWHRISV
2	4	TTTSLVLERSLNR	22	64	QKTTWHRISVFRP
3	7	SLVLERSLNRVHL	23	67	TWHRISVFRPGLR
4	10	LERSLNRVHLLGR	24	70	RISVFRPGLRDVA
5	13	SLNRVHLLGRVGQ	25	73	VFRPGLRDVAYQY
6	16	RVHLLGRVGQDPV	26	76	PGLRDVAYQYVKK
7	19	LLGRVGQDPVLRQ	27	79	RDVAYQYVKKGSR
8	22	RVGQDPVLRQVEG	28	82	AYQYVKKGSRIYL
9	25	QDPVLRQVEGKNP	29	85	YVKKGSRIYLEGK
10	28	VLRQVEGKNPVTI	30	88	KGSRIYLEGKIDY
11	31	QVEGKNPVTIFSL	31	91	RIYLEGKIDYGEY
12	34	GKNPVTIFSLATN	32	94	LEGKIDYGEYMDK
13	37	PVTIFSLATNEMW	33	97	KIDYGEYMDKNNV
14	40	IFSLATNEMWRSG	34	100	YGEYMDKNNVRRQ
15	43	LATNEMWRSGDSE	35	103	YMDKNNVRRQATT
16	46	NEMWRSGDSEVYQ	36	106	KNNVRRQATTIIA
17	49	WRSGDSEVYQLGD	37	109	VRRQATTIIADNI
18	52	GDSEVYQLGDVSQ	38	112	QATTIIADNIIFL
19	55	EVYQLGDVSQKTT	39	115	TIIADNIIFLSDQ
20	58	QLGDVSQKTTWHR	40	118	ADNIIFLSDQTKE
			41	120	NIIFLSDQTKEKE *

c

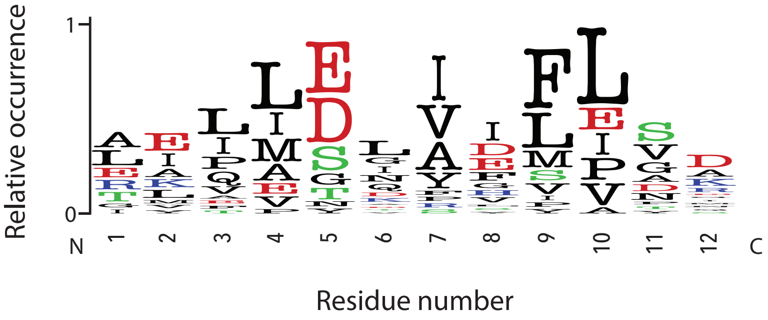

Supplementary Figure 4. Human CLPX ZBD binding to cellulose bound 13-mer peptides in mtSSB.

a. The ZBD of human CLPX interacts with 13-mer peptides in human mitochondrial single stranded DNA-binding protein (mtSSB). **b.** Spot numbering (and peptide sequences) for mtSSB peptide library (*a*, above) are indicated. **c**. Sequence logo (weblogo.berkeley.edu) was determined from alignment of all moderate to strong binding peptides identified in ***a*** (above) and Supplementary Figure 5.

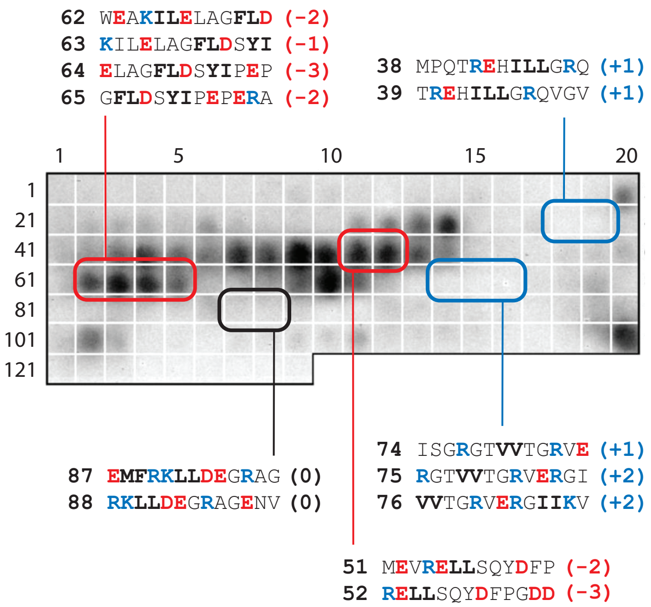

spot	aa	sequence	spot	aa	sequence	spot	aa	sequence
1	1	SKEKFERTKPHVN	46	136	KCDMVDDEELLEL	91	271	GENVGVLLRGIKR
2	4	KFERTKPHVNVGT	47	139	MVDDEELLELVEM	92	274	VGVLLRGIKREEI
3	7	RTKPHVNVGTIGH	48	142	DEELLELVEMEVR	93	277	LLRGIKREEIERG
4	10	PHVNVGTIGHVDH	49	145	LLELVEMEVRELL	94	280	GIKREEIERGQVL
5	13	NVGTIGHVDHGKT	50	148	LVEMEVRELLSQY	95	283	REEIERGQVLAKP
6	16	TIGHVDHGKTTLT	51	151	MEVRELLSQYDFP	96	286	IERGQVLAKPGTI
7	19	HVDHGKTTLTAAI	52	154	RELLSQYDFPGDD	97	289	GQVLAKPGTIKPH
8	22	HGKTTLTAAITTV	53	157	LSQYDFPGDDTPI	98	292	LAKPGTIKPHTKF
9	25	TTLTAAITTVLAK	54	160	YDFPGDDTPIVRG	99	295	PGTIKPHTKFESE
10	28	TAAITTVLAKTYG	55	163	PGDDTPIVRGSAL	100	298	IKPHTKFESEVYI
11	31	ITTVLAKTYGGAA	56	166	DTPIVRGSALKAL	101	301	HTKFESEVYILSK
12	34	VLAKTYGGAARAF	57	169	IVRGSALKALEGD	102	304	FESEVYILSKDEG
13	37	KTYGGAARAFDQI	58	172	GSALKALEGDAEW	103	307	EVYILSKDEGGRH
14	40	GGAARAFDQIDNA	59	175	LKALEGDAEWEAK	104	310	ILSKDEGGRHTPF
15	43	ARAFDQIDNAPEE	60	178	LEGDAEWEAKILE	105	313	KDEGGRHTPFFKG
16	46	FDQIDNAPEEKAR	61	181	DAEWEAKILELAG	106	316	GGRHTPFFKGYRP
17	49	IDNAPEEKARGIT	62	184	WEAKILELAGFLD	107	319	HTPFFKGYRPQFY
18	52	APEEKARGITINT	63	187	KILELAGFLDSYI	108	322	FFKGYRPQFYFRT
19	55	EKARGITINTSHV	64	190	ELAGFLDSYIPEP	109	325	GYRPQFYFRTTDV
20	58	RGITINTSHVEYD	65	193	GFLDSYIPEPERA	110	328	PQFYFRTTDVTGT
21	61	TINTSHVEYDTPT	66	196	DSYIPEPERAIDK	111	331	YFRTTDVTGTIEL
22	64	TSHVEYDTPTRHY	67	199	IPEPERAIDKPFL	112	334	TTDVTGTIELPEG
23	67	VEYDTPTRHYAHV	68	202	PERAIDKPFLLPI	113	337	VTGTIELPEGVEM
24	70	DTPTRHYAHVDCP	69	205	AIDKPFLLPIEDV	114	340	TIELPEGVEMVMP
25	73	TRHYAHVDCPGHA	70	208	KPFLLPIEDVFSI	115	343	LPEGVEMVMPGDN
26	76	YAHVDCPGHADYV	71	211	LLPIEDVFSISGR	116	346	GVEMVMPGDNIKM
27	79	VDCPGHADYVKNM	72	214	IEDVFSISGRGTV	117	349	MVMPGDNIKMVVT
28	82	PGHADYVKNMITG	73	217	VFSISGRGTVVTG	118	352	PGDNIKMVVTLIH
29	85	ADYVKNMITGAAQ	74	220	ISGRGTVVTGRVE	119	355	NIKMVVTLIHPIA
30	88	VKNMITGAAQMDG	75	223	RGTVVTGRVERGI	120	358	MVVTLIHPIAMDD
31	91	MITGAAQMDGAIL	76	226	VVTGRVERGIIKV	121	361	TLIHPIAMDDGLR
32	94	GAAQMDGAILVVA	77	229	GRVERGIIKVGEE	122	364	HPIAMDDGLRFAI
33	97	QMDGAILVVAATD	78	232	ERGIIKVGEEVEI	123	367	AMDDGLRFAIREG
34	100	GAILVVAATDGPM	79	235	IIKVGEEVEIVGI	124	370	DGLRFAIREGGRT
35	103	LVVAATDGPMPQT	80	238	VGEEVEIVGIKET	125	373	RFAIREGGRTVGA
36	106	AATDGPMPQTREH	81	241	EVEIVGIKETQKS	126	376	IREGGRTVGAGVV
37	109	DGPMPQTREHILL	82	244	IVGIKETQKSTCT	127	379	GGRTVGAGVVAKV
38	112	MPQTREHILLGRQ	83	247	IKETQKSTCTGVE	128	381	RTVGAGVVAKVLS *
39	115	TREHILLGRQVGV	84	250	TQKSTCTGVEMFR			
40	118	HILLGRQVGVPYI	85	253	STCTGVEMFRKLL			
41	121	LGRQVGVPYIIVF	86	256	TGVEMFRKLLDEG			
42	124	QVGVPYIIVFLNK	87	259	EMFRKLLDEGRAG			
43	127	VPYIIVFLNKCDM	88	262	RKLLDEGRAGENV			
44	130	IIVFLNKCDMVDD	89	265	LDEGRAGENVGVL			
45	133	FLNKCDMVDDEEL	90	268	GRAGENVGVLLRG			

Supplementary Figure 5. Human CLPX ZBD binding to cellulose bound 13-mer peptides in EFTu.

The ZBD of human CLPX interacts with 13-mer peptides in *E. coli* elongation factor thermo unstable (*ec*EFTu, upper panel). Strong binding peptides contain one or more hydrophobic patches and exhibit a net negative charge (highlighted in red). Non-binding peptides containing a hydrophobic patch and a net positive charge are indicated (blue), while non-binding peptides containing a hydrophobic patch and no net charge are indicated (black). Spot numbering and sequences are indicated in the lower panel.

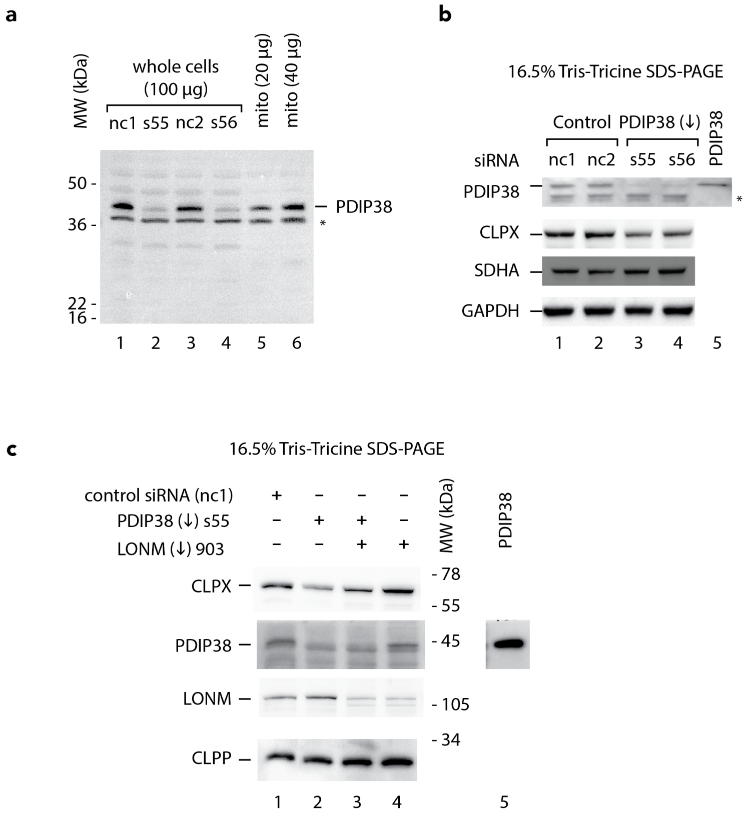

Supplementary Figure 6. The steady state levels of CLPX are reduced in cells lacking PDIP38.

a. The steady state levels of PIDP38 were analysed in HeLa cells 72 hours post-transfection with either Silencer Select Negative Control No. 1 siRNA (nc1, lane 1) and Negative Control No. 2 (nc2, lane 3) and compared to HeLa cells treated with *PDIP38*-targeted Silencer Select siRNAs s25055 (s55, lane 2) and s25056 (s56, lane 4) and compared to PDIP38 levels in 20 µg (lane 5) or 40 µg (lane 6) crude mitochondria (mito). **b.** The steady state levels of PDIP38 (top panel), CLPX (2nd panel) SDHA (3rd panel) and GAPDH (bottom panel) were analysed in HeLa cells 72 hours post-transfection with either Silencer Select Negative Control No. 1 siRNA (nc1, lane 1) and Negative Control No. 2 (nc2, lane 2) and compared to HeLa cells treated with *PDIP38*-targeted Silencer Select siRNAs s25055 (s55, lane 3) and s25056 (s56, lane 4). Lane 5, purified untagged PDIP38. **c.** The steady state levels of CLPX (top panel), PDIP38 (2nd panel) LONM (3rd panel) and CLPP (bottom panel) were analysed in HeLa cells 72 hours post-transfection with either Silencer Select Negative Control No. 1 siRNA (nc1, lane 1), s25055 for targeted knock down of PDIP38 (s55, lane 2), siRNA 903 for targeted knock down of LONM (lane 4) siRNA s25055 and siRNA 903 for targeted knock down of both PDIP38 and LONM (lane 3). (**a-c**) Proteins were separated by 16.5% Tris-Tricine SDS-PAGE and subjected to immunoblotting with the appropriate antisera to visualize endogenous proteins. (*, non-specific cross-reactivity of PDIP38 antisera, lower band on separation using 16.5% Tris-Tricine SDS-PAGE).

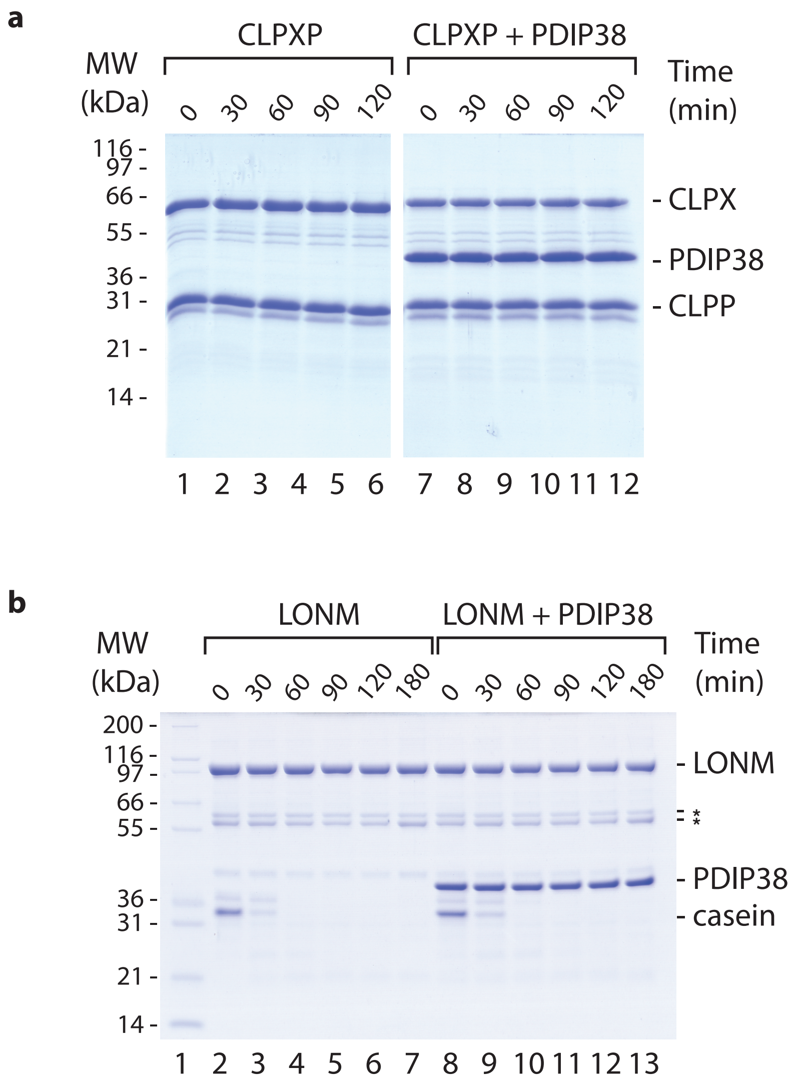

Supplementary Figure 7. Effect of PDIP38 on CLPXP and LONM

a. The potential for CLPXP to facilitate the autocatalytic turnover of CLPX was examined *in vitro* in the absence (lane 1 – 6) or presence of 1 μM PDIP38 (lanes 7 – 12). **b**. *In vitro* degradation of casein by $LONM_6$ protease (400 nM) in the absence (lane 2 – 7) or presence of 1 μM PDIP38 (lanes 8 – 13). All proteins were separated by 10% Tris-Tricine SDS-PAGE and visualised by CBB staining.

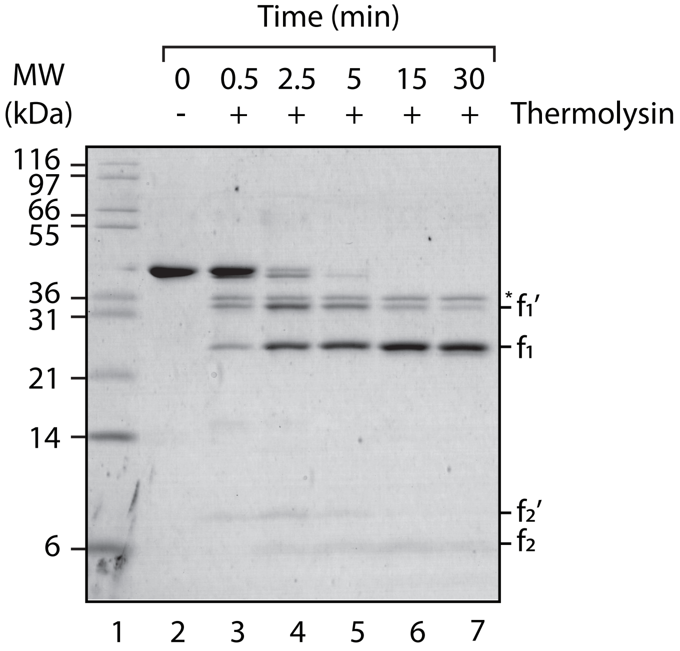

Supplementary Figure 8. Human PDIP38 is composed of two structural domains.

Limited proteolysis of native His_{10}-tagged PDIP38 using thermolysin. Samples were analyzed by Coomassie stained 16.5% Tricine-buffered SDS-PAGE. *, thermolysin. f1, f1', f2, f2', fragments of PDIP38.

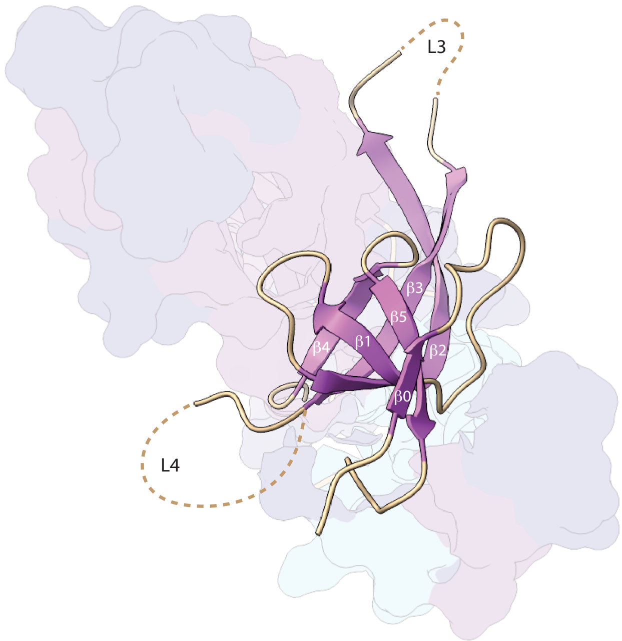

Supplementary Figure 9. Structure of PDIP38 yccV-like domain, relative to DUF525 domain

Ribbon representation of human PDIP38 N-terminal yccV-like domain highlighting the β-strands (purple) and loops (tan), with C-terminal domain shown in surface representation. Loop 3 (L3) and loop 4 (L4) are disordered and hence represented by a dotted line. The extended β2 and β3 strands form a continuous sheet with the top sheet of the immunoglobulin-like fold of the DUF525 domain. The figure was generated in ChimeraX_Daily.

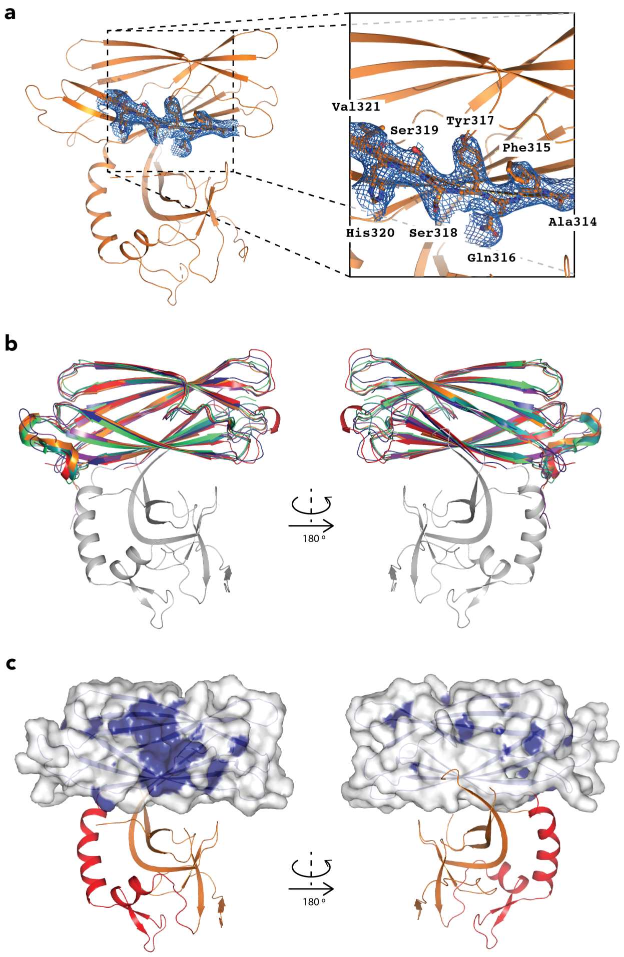

Supplementary Figure 10. Structural alignment of PDIP38$_{DUF525}$ with Fbxo3$_{DUF525}$ and bactierial ApaG proteins, highlighting the conserved groove.

(**a**) Electron density map 2Fo-Fc calculated at 1 sigma (shown in blue) surrounding the residues 314-321 of β13 in 6ZLX. Left picture displays the entire structure in cartoon representation while the right picture is a close-up of β13 located on the C-terminal DUF525 domain. (**b-c**) Structural comparison of human PDIP38 DUF525 domain (blue), aligned with the DUF525 domain of Fbxo3 (PDB: 5HDW [1], red) and ApaG proteins from Xanthomonas citri (PDB: 2F1E [2], lime green), Bordetella pertusis (PDB: 1XQ4, pale green), Vibrio cholerae (PDB: 1XVS, orange) and Shewanella ondeiensis (PDB: 1TZA, purple) in **(b)** ribbon representation and **(c)** surface representation shown in front (right) and back (left) view. The back view highlights the conserved hydrophobic residues (blue) that line the putative substrate binding groove of the DUF525 domain. In (b) the N-terminal region of PDIP38 is shown in grey, while in (c) the N-terminal region of PDIP38 is shown in orange (YccV-like domain) and red (linker region).

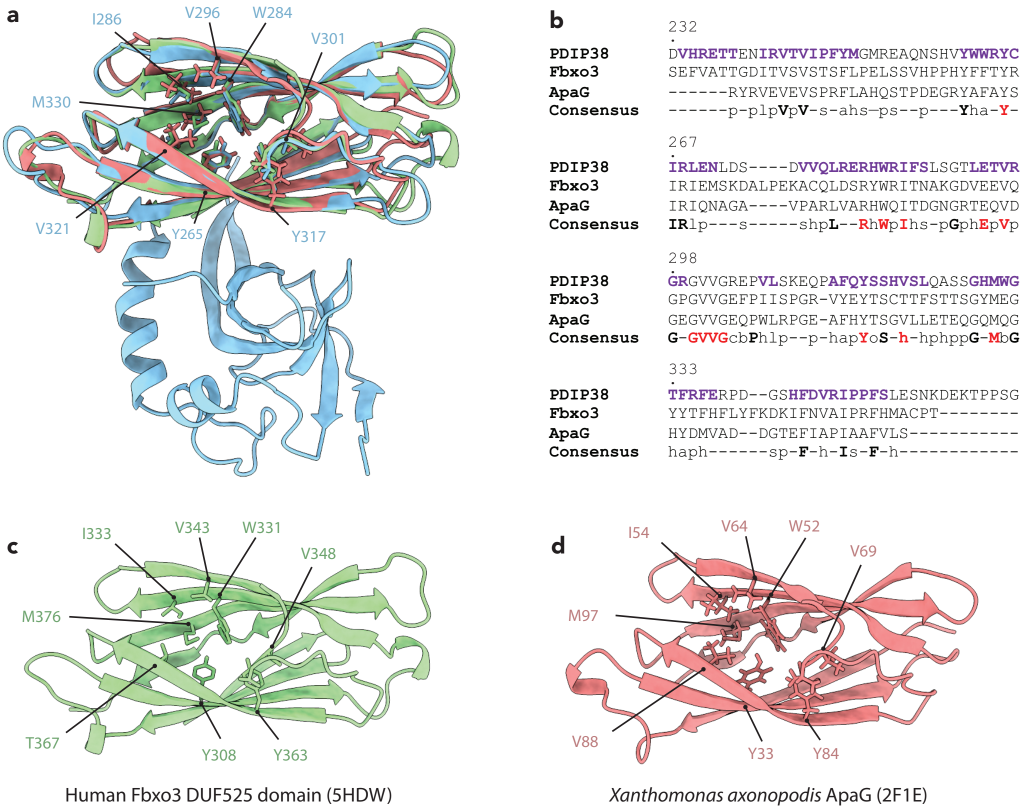

Supplementary Figure 11. Structural alignment of PDIP38 with Fbxo3$_{DUF525}$ and ApaG highlighting the conserved residues located in the putative substrate binding pocket .

a. Ribbon representation of human PDIP38 (pale blue), aligned with the DUF525 domain of Fbxo3 (green) and ApaG (red) illustrating the conserved hydrophobic residues that line the putative substrate binding groove. **b.** Protein sequence alignment of human PDIP38 illustrating residues that are absolutely conserved (bold) and located within the hydrophobic pocket (red). h, hydrophobic residue. **c.** Ribbon representation of DUF525 domain of Fbxo3 (green). **d**. Ribbon representation of DUF525 domain of ApaG (red).

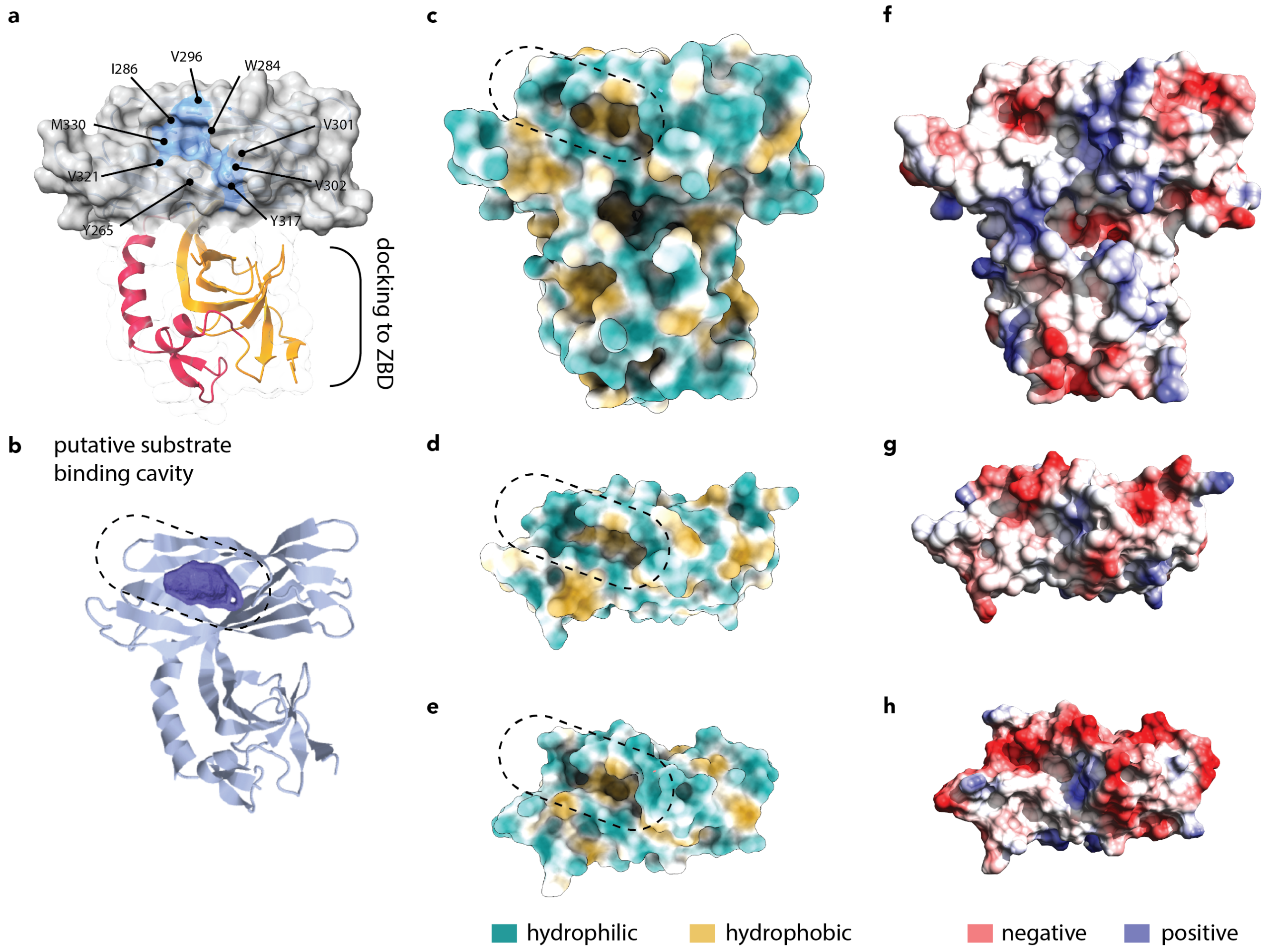

Supplementary Figure 12. Surface composition of PDIP38, Fbxo3$_{DUF525}$ and ApaG.

a-b. Ribbon representation of PDIP38 showing (a) the surface of the DUF525 (light grey) highlighting the conserved hydrophobic residues (blue) that line the proposed substrate binding groove of Fbxo3 (as described in Supplementary Table 1, Supplementary Figures 11 and 12) and the relative position of the N-terminal YccV-like domain (for docking to the ZBD of CLPX) and (b) highlighting the putative substrate binding pocket (as defined by CavityPlus [3]), located in the C-terminal DUF525 domain. (c-h) Comparison of the hydrophobic (**c-e**) and electrostatic (**f-h**) surfaces of human PDIP38 (**c** and **f**), human Fbxo3 (**d** and **g**) and *Xanthomonas citri* ApaG (**e** and **h**). Green – hydrophilic, yellow, hydrophobic, red – negatively charged and blue – positively charged.

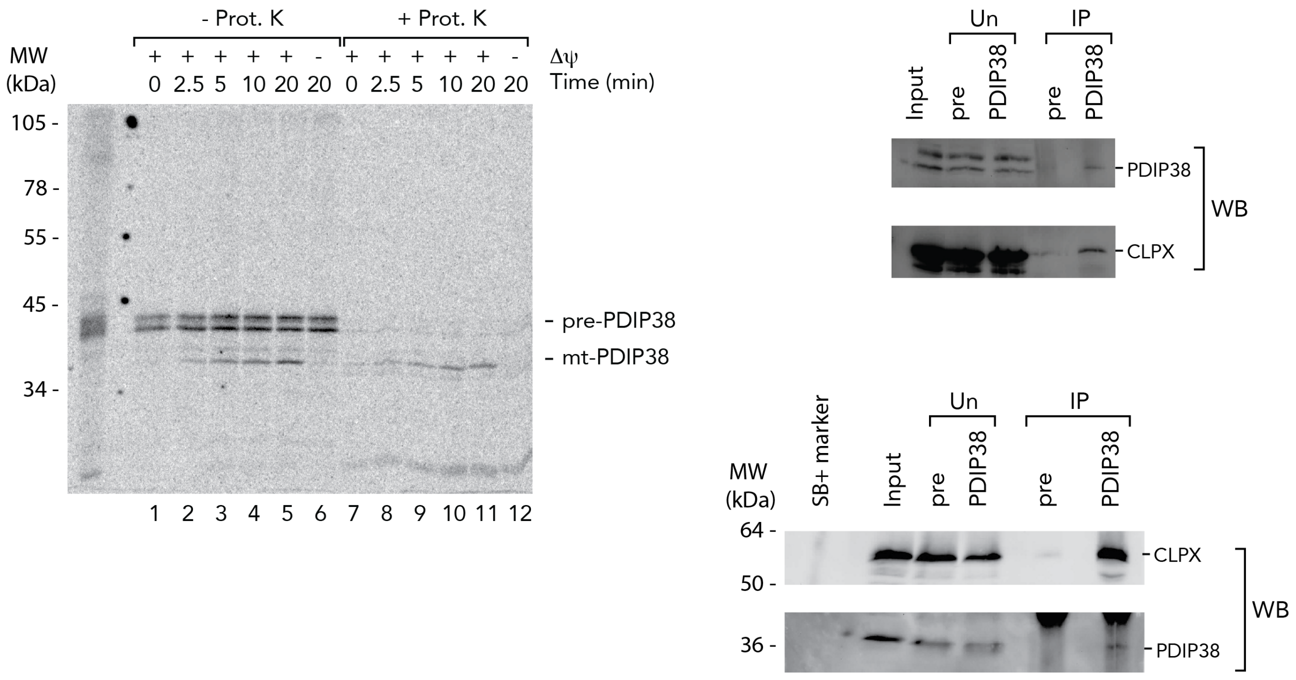

Supplementary Figure 13. Full length autoradiograph and immunoblot strips from Figure 1.

Full length gel digital autoradiography of Figure 1a. Full immunoblot strips from Figure 1c and d.

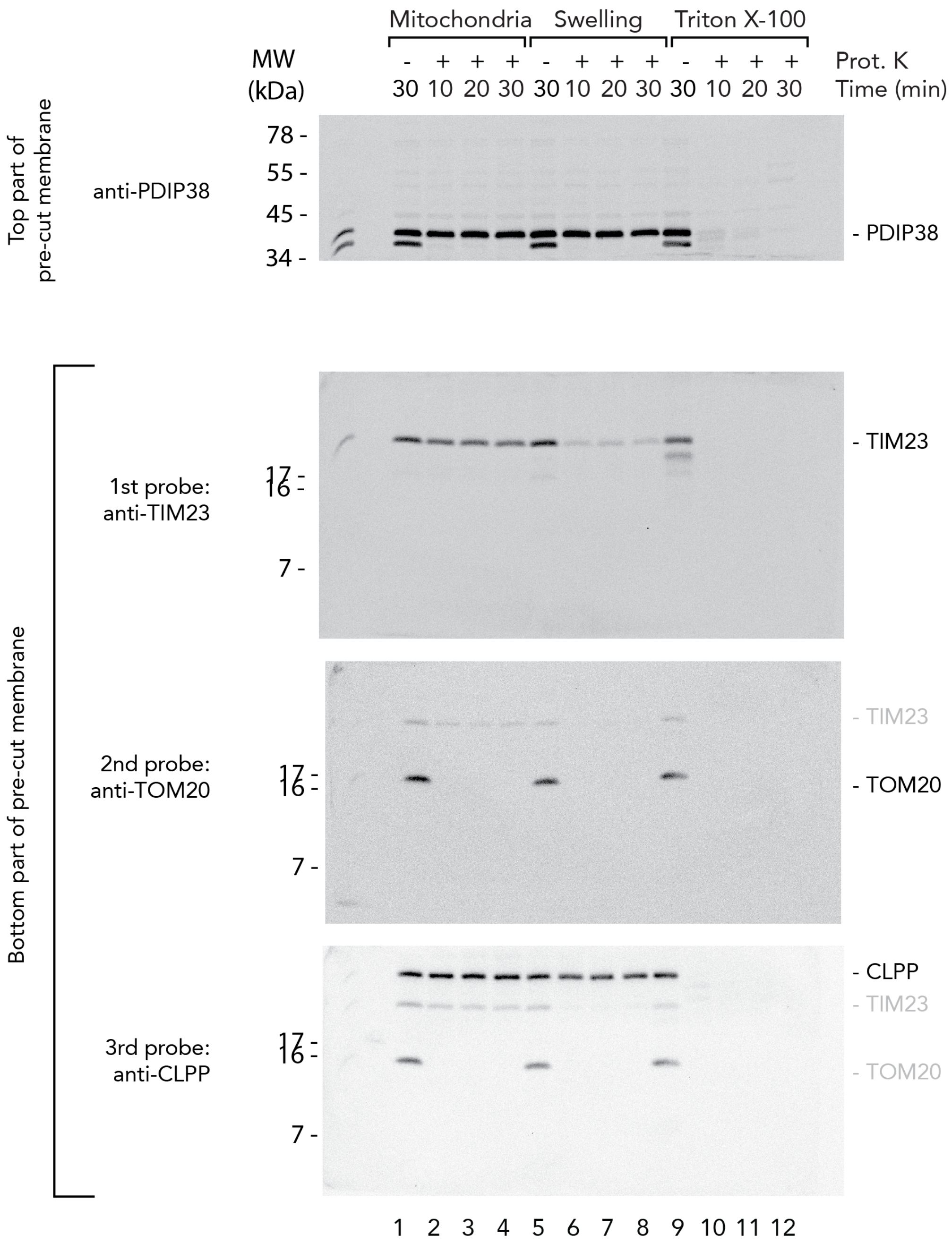

Supplementary Figure 14. Full length immunoblot strips from Figure 1b.

Full immunoblot strips from Figure 1b.

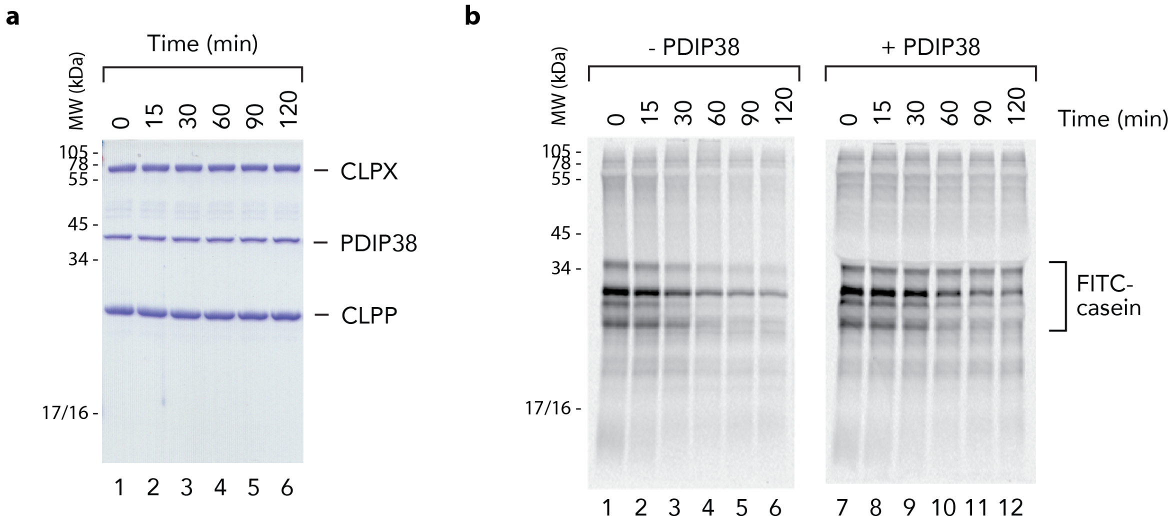

Supplementary Figure 15. Full length gel images from Figure 2b.

Full length gel images from Figure 2b.

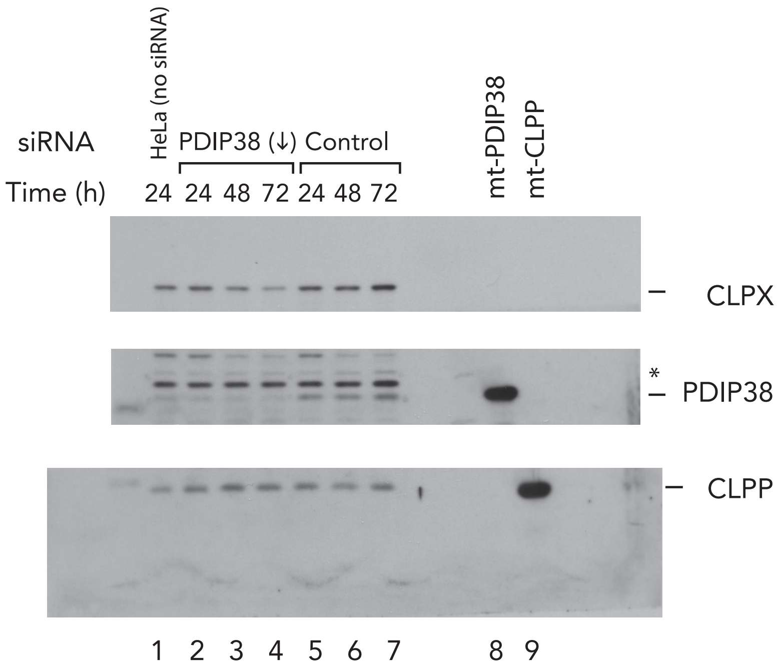

Supplementary Figure 16. Full length gel images from Figure 4a.

Full length gel images from Figure 4a.

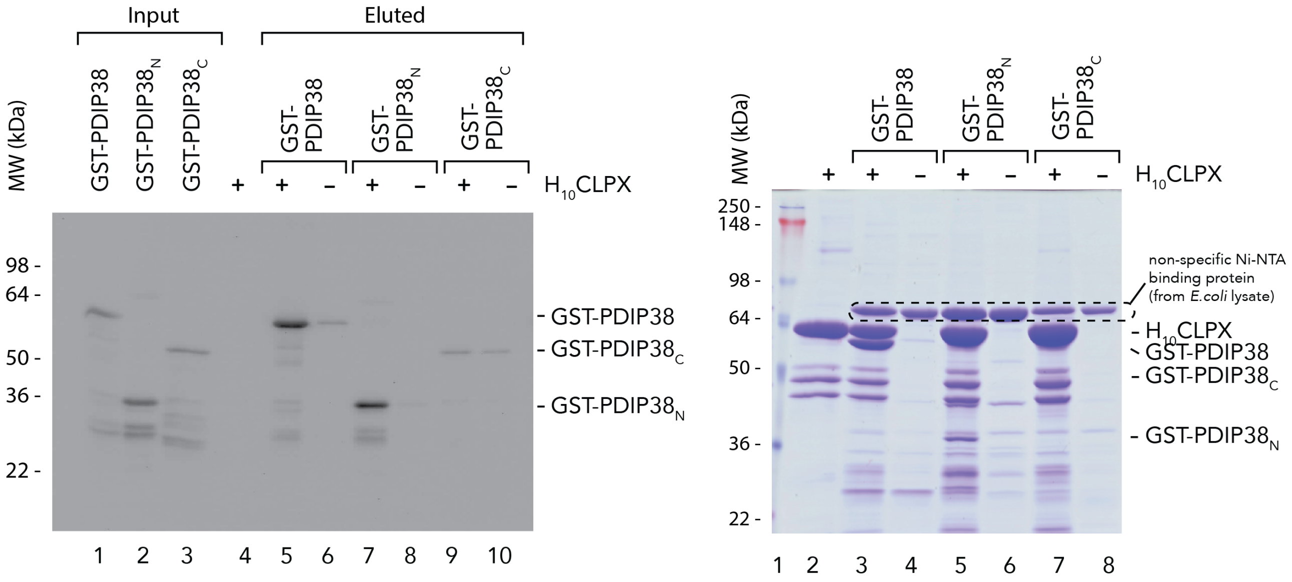

Supplementary Figure 17. Full length gel image and immunoblot from Figure 5b.

Full length gel image and immunoblot from Figure 5b.

References

1. Krzysiak TC, Chen BB, Lear T, Mallampalli RK, Gronenborn AM. Crystal structure and interaction studies of the human FBxo3 ApaG domain. *FEBS J* **283**, 2091-2101 (2016).

2. Cicero DO*, et al.* Solution structure of ApaG from Xanthomonas axonopodis pv. citri reveals a fibronectin-3 fold. *Proteins* **67**, 490-500 (2007).

3. Xu Y*, et al.* CavityPlus: a web server for protein cavity detection with pharmacophore modelling, allosteric site identification and covalent ligand binding ability prediction. *Nucleic Acids Res* **46**, W374-W379 (2018).

4 Lowth, B. R. *et al.* Substrate recognition and processing by a Walker B mutant of the human mitochondrial AAA+ protein CLPX. *J Struct Biol* **179**, 193-201, doi:10.1016/j.jsb.2012.06.001 (2012).

5 Dougan, D. A., Weber-Ban, E. & Bukau, B. Targeted delivery of an ssrA-tagged substrate by the adaptor protein SspB to its cognate AAA+ protein ClpX. *Mol Cell* **12**, 373-380 (2003).

6 Bezawork-Geleta, A., Saiyed, T., Dougan, D. A. & Truscott, K. N. Mitochondrial matrix proteostasis is linked to hereditary paraganglioma: LON-mediated turnover of the human flavinylation factor SDH5 is regulated by its interaction with SDHA. *FASEB J* **28**, 1794-1804, doi:10.1096/fj.13-242420 (2014).
