# Supplementary material for: Polymerase delta-interacting protein 38 (PDIP38) modulates the stability and activity of the mitochondrial AAA+ protease CLPXP
Source: Commun Biol. 2020 Nov 6;3:646. doi: 10.1038/s42003-020-01358-6 (PMC7647994; doi:10.1038/s42003-020-01358-6)
Supplement: Supplementary file 3 — Description of Additional Supplementary Files [file 42003_2020_1358_MOESM3_ESM.pdf]

Description of Additional Supplementary Files

File Name: Supplementary Data 1
Description: Source data behind graphs in Figure 4a and 4c.
