# Supplementary material for: Polymerase delta-interacting protein 38 (PDIP38) modulates the stability and activity of the mitochondrial AAA+ protease CLPXP
Source: Commun Biol. 2020 Nov 6;3:646. doi: 10.1038/s42003-020-01358-6 (PMC7647994; doi:10.1038/s42003-020-01358-6)
Supplement: Supplementary file 5 — Reporting Summary [file 42003_2020_1358_MOESM5_ESM.pdf]

nature research

Corresponding author(s): David A. Dougan, Kaye N. Truscott

Last updated by author(s): Sep 23, 2020

Reporting Summary

Nature Research wishes to improve the reproducibility of the work that we publish. This form provides structure for consistency and transparency in reporting. For further information on Nature Research policies, see our Editorial Policies and the Editorial Policy Checklist.

Statistics

For all statistical analyses, confirm that the following items are present in the figure legend, table legend, main text, or Methods section.

n/a	Confirmed	
☐	☒	The exact sample size (n) for each experimental group/condition, given as a discrete number and unit of measurement
☐	☒	A statement on whether measurements were taken from distinct samples or whether the same sample was measured repeatedly
☒	☐	The statistical test(s) used AND whether they are one- or two-sided<br>*Only common tests should be described solely by name; describe more complex techniques in the Methods section.*
☒	☐	A description of all covariates tested
☒	☐	A description of any assumptions or corrections, such as tests of normality and adjustment for multiple comparisons
☐	☒	A full description of the statistical parameters including central tendency (e.g. means) or other basic estimates (e.g. regression coefficient) AND variation (e.g. standard deviation) or associated estimates of uncertainty (e.g. confidence intervals)
☒	☐	For null hypothesis testing, the test statistic (e.g. F, t, r) with confidence intervals, effect sizes, degrees of freedom and P value noted<br>*Give P values as exact values whenever suitable.*
☒	☐	For Bayesian analysis, information on the choice of priors and Markov chain Monte Carlo settings
☒	☐	For hierarchical and complex designs, identification of the appropriate level for tests and full reporting of outcomes
☒	☐	Estimates of effect sizes (e.g. Cohen's d, Pearson's r), indicating how they were calculated

Our web collection on statistics for biologists contains articles on many of the points above.

Software and code

Policy information about availability of computer code

Data collection	*Provide a description of all commercial, open source and custom code used to collect the data in this study, specifying the version used OR state that no software was used.*
Data analysis	*Provide a description of all commercial, open source and custom code used to analyse the data in this study, specifying the version used OR state that no software was used.*

For manuscripts utilizing custom algorithms or software that are central to the research but not yet described in published literature, software must be made available to editors and reviewers. We strongly encourage code deposition in a community repository (e.g. GitHub). See the Nature Research guidelines for submitting code & software for further information.

Data

Policy information about availability of data

All manuscripts must include a data availability statement. This statement should provide the following information, where applicable:

- Accession codes, unique identifiers, or web links for publicly available datasets
- A list of figures that have associated raw data
- A description of any restrictions on data availability

Data supporting the findings of this manuscript are available from the corresponding author upon reasonable request. A reporting summary for this article is available as a Supplementary Information file. Atomic coordinates for the PDIP38 structure have been deposited in wwPDB under accession codes PDB 6ZLX.

Field-specific reporting

Please select the one below that is the best fit for your research. If you are not sure, read the appropriate sections before making your selection.

- [x] Life sciences
- [ ] Behavioural & social sciences
- [ ] Ecological, evolutionary & environmental sciences

For a reference copy of the document with all sections, see nature.com/documents/nr-reporting-summary-flat.pdf

Life sciences study design

All studies must disclose on these points even when the disclosure is negative.

Sample size	No statistical methods were used to predetermine sample sizes.
Data exclusions	No data were excluded
Replication	Each assay was performed at least twice. All attempts at replication were successful
Randomization	N/A
Blinding	N/A

Reporting for specific materials, systems and methods

We require information from authors about some types of materials, experimental systems and methods used in many studies. Here, indicate whether each material, system or method listed is relevant to your study. If you are not sure if a list item applies to your research, read the appropriate section before selecting a response.

Materials & experimental systems

n/a	Involved in the study	
[ ]	[x]	Antibodies
[ ]	[x]	Eukaryotic cell lines
[x]	[ ]	Palaeontology and archaeology
[x]	[ ]	Animals and other organisms
[x]	[ ]	Human research participants
[x]	[ ]	Clinical data
[x]	[ ]	Dual use research of concern

Methods

n/a	Involved in the study	
[x]	[ ]	ChIP-seq
[x]	[ ]	Flow cytometry
[x]	[ ]	MRI-based neuroimaging

Antibodies

Antibodies used	polyclonal Anti-POLDIP2 (PDIP38) antisera raised in goat (abcam catalog number ab109805), polyclonal Anti-CLPX antisera raised in rabbit ;polyclonal Anti-LONM antisera raised in rabbit; monoclonal anti-TIM23 antibody (BD Biosciences catalog number 611223) raised in mouse. Monoclonal anti-SDHA antibody (Invitrogen catalog number 459200) raised in mouse. polyclonal Anti-GST (GE Healthcare catalog number 27-4577-01) raised in goat. monoclonal Anti-GAPDH (Life Technologies catalog number AM4300) raise in mouse.
Validation	Anti-POLDIP2 (PDIP38) antibody raised in goat against amino acids 129-143 of Human POLDIP2 (NP_056399.1), validated by comparison to PDIP38 KD; Anti-CLPX and Anti-LONM antisera validated in Lowth et al., (2012) by comparison to KD of CLPX or LONM, respectively. Anti-GST validated with purified protein. Anti-SDHA antibody validated by KD.

Eukaryotic cell lines

Policy information about cell lines

Cell line source(s)	HeLa cells from ATTC
Authentication	HeLa cells were authenticated by STR profiling at Cellbank Australia
Mycoplasma contamination	HeLa tested negative for mycoplasma contamination.
Commonly misidentified lines (See ICLAC register)	N/A
